# ﻿*Scorpiopslourencoi* sp. nov., the revalidation of *Scorpiopsatomatus* Qi, Zhu & Lourenço, 2005, and the redescription of *Scorpiopstibetanus* Hirst, 1911 (Scorpiones, Scorpiopidae) from China

**DOI:** 10.3897/zookeys.1132.87364

**Published:** 2022-11-30

**Authors:** Heyu Lv, Zhiyong Di

**Affiliations:** 1 Key Laboratory of Zoological Systematics and Application of Hebei Province, College of Life Sciences, Hebei University, Baoding, Hebei 071002, China Hebei University Baoding China; 2 Institute of Life Sciences and Green Development, Hebei University, Baoding 071002, Hebei, China Hebei University Baoding China

**Keywords:** China, new species, *Scorpiops*, Xizang

## Abstract

*Scorpiopslourencoi***sp. nov.** is described from Shigatse, Xizang. *Scorpiopsatomatus* Qi, Zhu & Lourenço, 2005 and *Scorpiopspococki* Qi, Zhu & Lourenço, 2005 were synonymized by Kovařík et al. (2020) as two junior synonyms of *Scorpiopstibetanus* Hirst, 1911 but based on several field surveys in Xizang in recent years, and a careful survey of the literature, *S.atomatus* is reaffirmed as a valid species and *S.tibetanus* is redescribed, both based on newly collected specimens. This brings the total number of species of *Scorpiops* recorded in China to 32.

## ﻿Introduction

Kovařík et al. (2020) and Šťáhlavský et al. (2020) revised the family Scorpiopidae Kraepelin, 1905 and synonymized the previously accepted genera of the family Scorpiopidae, excepting *Parascorpiops* Banks, 1928, with the genus *Scorpiops* Peters, 1861. Lourenço and Ythier (2022) reclassified the genera of the family Scorpiopidae, proposed six subgenera of the genus *Scorpiops* (including *Alloscorpiops* Vachon, 1980, *Dasyscorpiops* Vachon, 1974, *Euscorpiops* Vachon, 1980, *Neoscorpiops* Vachon, 1980, *Plethoscorpiops* Lourenço, 2017 and *Scorpiops*), and maintained *Parascorpiops* as a distinct genus of *Scorpiops*.

*Scorpiops* is the main member of the family Scorpiopidae, distributed mainly in south and southeast Asia, and currently includes 104 species (Lourenço and Ythier 2022; Tang 2022). In China, thirty species are found in Hubei, Xinjiang, Xizang, and Yunnan (Lv and Di 2022; Tang 2022).

Hirst (1911) erected *S.tibetanus*, based on specimens from “Tsangpo Valley, Chaksam Ferry”, Xizang. This appears to be the first species of the genus *Scorpiops* found in China. Kovařík (2000) reported new additions to the distribution range of *S.tibetanus*: Kambu basti, Lhasa, and Shigatse, and provided outline drawings of the chelae of both sexes. The figures are not accurate, but the remarkable thickness, nearly square, and distinctly rectangular dimorphism of the chelae of both sexes is clear. Kovařík (2000) examined the holotype (male) and collected new materials of *S.tibetanus*, recording some important new information: the ventral trichobothria on the patella number 7–10 (but usually 9; in one young of 37 specimens, 7 on one side).

Qi et al. (2005) reported one small species, *S.atomatus*, and two medium species, *Scorpiopslangxian* Qi, Zhu & Lourenço, 2005 and *S.pococki* and one large species, *Scorpiopsluridus* Qi, Zhu & Lourenço, 2005. The type locality of *S.atomatus* is Lang County, and other localities include Chayu and Gyaca counties. The type locality of *S.pococki* is Gyaca County, and other localities include Chayu and Nêdong counties, Lhasa, Nyingchi, and Shigatse cities.

Kovařík and Ahmed (2009) provided a list of taxa of the *S.hardwickii* (Gervais, 1843) complex, which contains 12 species, widely distributed in Asia, including five species distributed in China: *S.atomatus*, *S.hardwickii*, *S.langxian*, *S.pococki*, and *S.tibetanus*. However, following the reports of Kovařík (2000), Kovařík and Ahmed (2009), Qi et al. (2005), and Di et al. (2011) thought that *S.atomatus* and *S.tibetanus* should be excluded from *S.hardwickii* complex. Di et al. (2013) described the female of *S.tibetanus* and revised the key for Chinese *Scorpiops* species. Kovařík et al. (2020) proposed *S.atomatus* and *S.pococki* as two junior synonyms of *S.tibetanus* and provided figures of freshly collected *S.tibetanus*.

In this work, we analyze species information from the literature in tandem with newly collected material, and confirm that *S.atomatus* is a valid species; additionally, we redescribe *S.tibetanus* based on new material and describe *S.lourencoi* as a new species.

## ﻿Materials and methods

Specimens were collected by hand and preserved in 75% ethanol. Type series of the new species are deposited in the Museum of Hebei University, Baoding, China (**MHBU**).

Illustrations and measurements were produced using a Leica M205 stereomicroscope. The photographs were taken with a Canon 650D camera and a Leica M205FA stereomicroscope (with a digital color microscope camera Leica DFC495). Measurements (in mm) follow Sissom (1990). Trichobothrial notations are done according to Vachon (1974), and the morphological terminology mostly follows Hjelle (1990). The terminology of metasomal carination is that of Vachon (1952), and the terminology of pedipalp chelal carinae follows Soleglad and Sissom (2001).

Movable finger dentition abbreviations used in the text are as follows: **ID**, inner denticles; **IAD**, inner accessory denticles; **MD**, median denticles; **OD**, outer denticles.

## ﻿Taxonomic treatment

### Family Scorpiopidae Kraepelin, 1905

#### ﻿Genus Scorpiops

Taxon classificationAnimaliaScorpionesEuscorpiidae

Peters, 1861

145D990D-D2B6-55F3-B0D1-EEA5B38F371A

##### Type species.

*Scorpiopshardwickii* Gervais, 1843.

##### Type locality.

India Himalaya.

#### ﻿ Scorpiops atomatus

Taxon classificationAnimaliaScorpionesEuscorpiidae

Qi, Zhu & Lourenço, 2005

C345DBE0-47F0-5FB3-9815-AD839BD9F9FF

Figs 1–4 , 5–14 , 15–24 , 25–32 , Table 1

Scorpiops atomatus Qi, Zhu & Lourenço, 2005: 6, 10, figs 16–31; Kovařík and Ahmed 2009: 10; Di et al. 2013: 59–61, figs 1–21, tab. 1; Di et al. 2014: 11.

##### Type locality.

China, Xizang, Lang County (29.02°N, 93.08°E).

##### Material examined.

2 males and 3 females, China, Xizang (Tibet), Linzhi City (Nyingchi City), Lang County (Nang County), Dongga Town (Tonga Town), 06/5/2017, Zhiyong Di leg, (Ar.-MHBU-ScXZLX17050601, 01–05).

##### Diagnosis

(modified from Qi et al. 2005). Adult body length 35–45 mm. Base color uniformly brown. Patella of pedipalp with 17 (5 *eb*, 2 *esb*, 2 *em*, 4 *est*, 4 *et*) external and 8–10 (usually nine) ventral trichobothria. Chelal trichobothria *Eb_3_* is located in proximal half of manus between trichobothria *Dt* and *Db.* Chela with four ventral trichobothria. Chela with an average length/width ratio of 2.3 in males (*n* = 2 adults) and 2.5 in females (*n* = 3 adults); pedipalp movable finger with ca. five ID, eight or nine IAD, 58–62 MD, and 7–9 OD present, chela fingers on adult males and females scalloped, usually more strongly in male. Pectinal teeth count 10–11 in males (*n* = 2 adults) and eight or nine in females (*n* = 3 adults), fulcra present. Pectinal with three marginal and six middle lamellae. Telson bulbous and granulate, annular ring absent.

**Figures 1–4.**
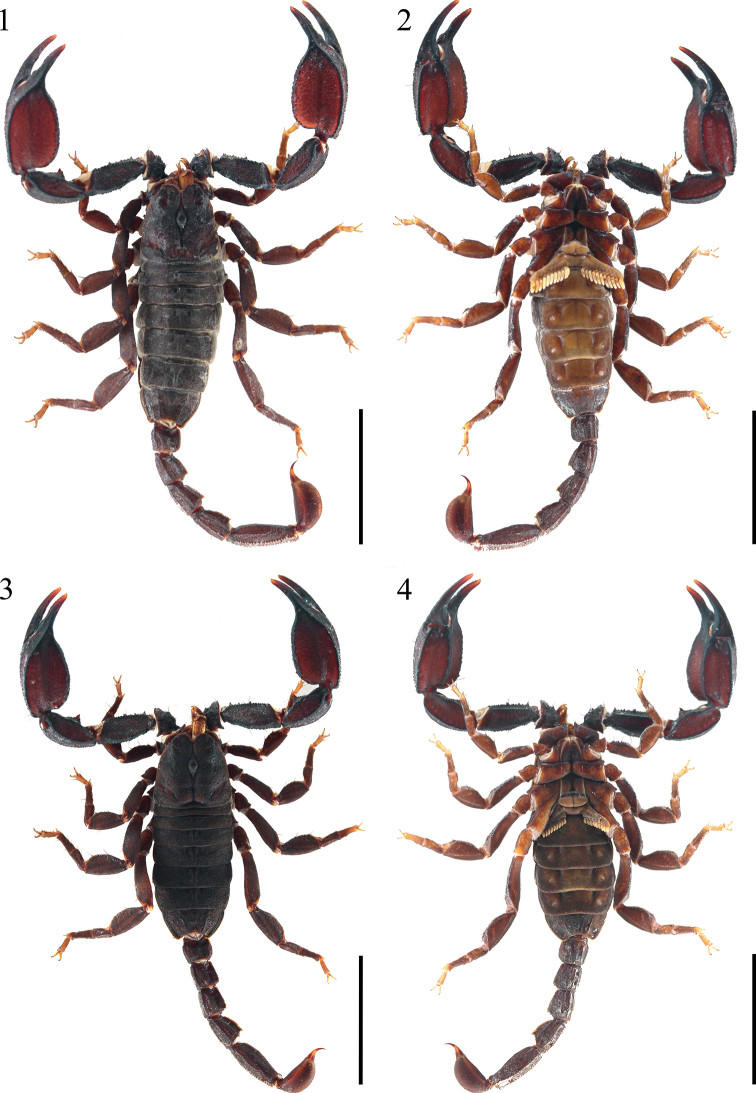
*Scorpiopsatomatus* from Lang County **1, 2** male (Ar.-MHBU-ScXZLX1705060101), dorsal and ventral views **3, 4** female (Ar.-MHBU-ScXZLX1705060102), dorsal and ventral views. Scale bars: 12.0 mm.

**Figures 5–14.**
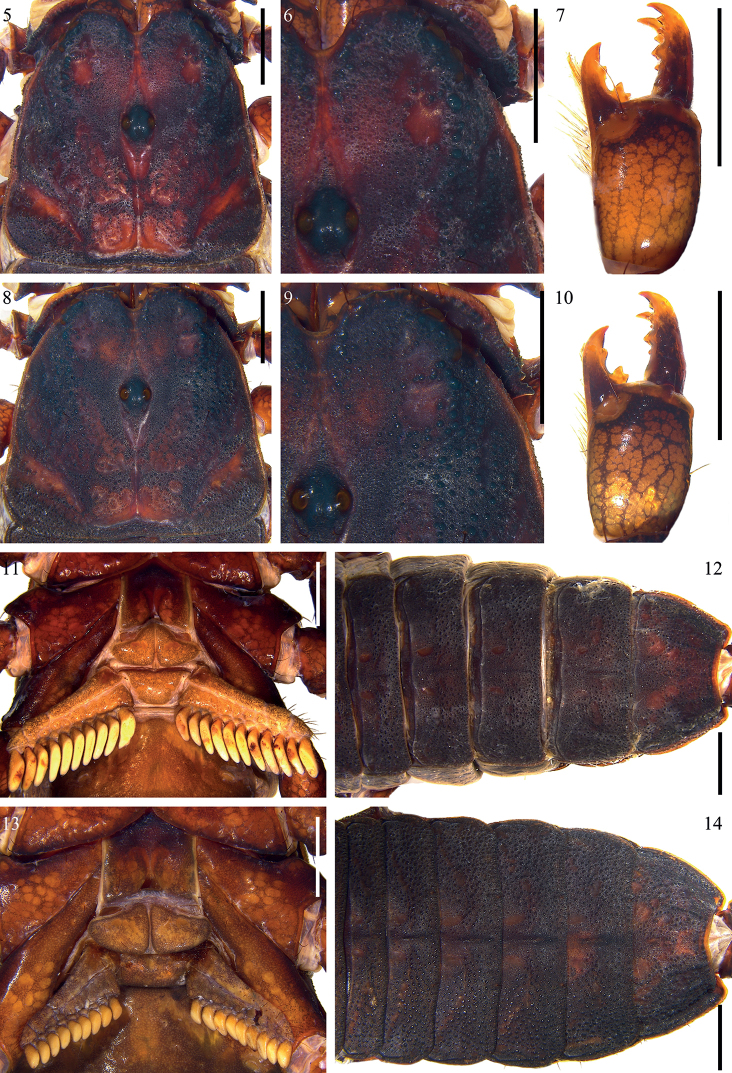
*Scorpiopsatomatus* from Lang County **5, 6, 7, 11, 12** male (Ar.-MHBU-ScXZLX1705060101) **8, 9, 10, 13, 14** female (Ar.-MHBU-ScXZLX1705060102) **5, 8** carapace **6, 9** eyes and nearby area **7, 10** chelicera dorsal surface **11, 13** sternum, genital operculum, and pectines **12, 14** tergites. Scale bars: 2.0 mm.

**Figures 15–24.**
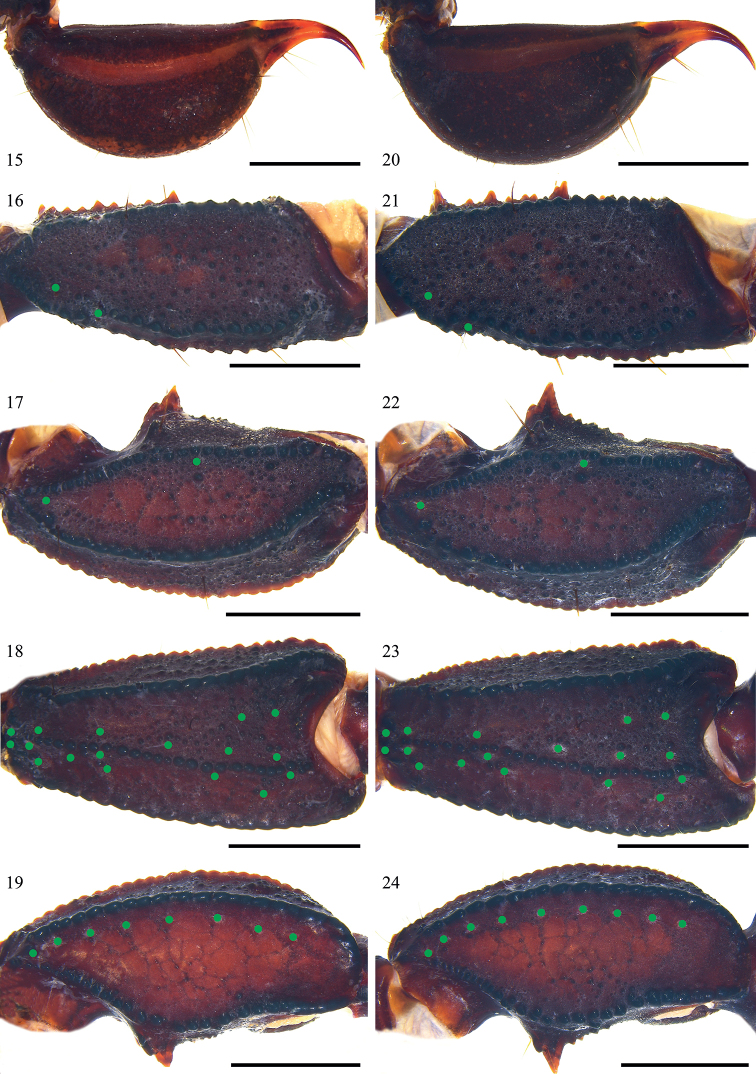
*Scorpiopsatomatus* from Lang County. **15–19** male (Ar.-MHBU-ScXZLX1705060101) **20–24** female (Ar.-MHBU-ScXZLX1705060102) **15, 20** telson, lateral surface **16, 21** femur dorsal surface **17–19, 22–24** patella dorsal, external, and ventral surfaces. Green dots showing trichobothrial patterns of pedipalps. Scale bars: 2.0 mm.

**Figures 25–32.**
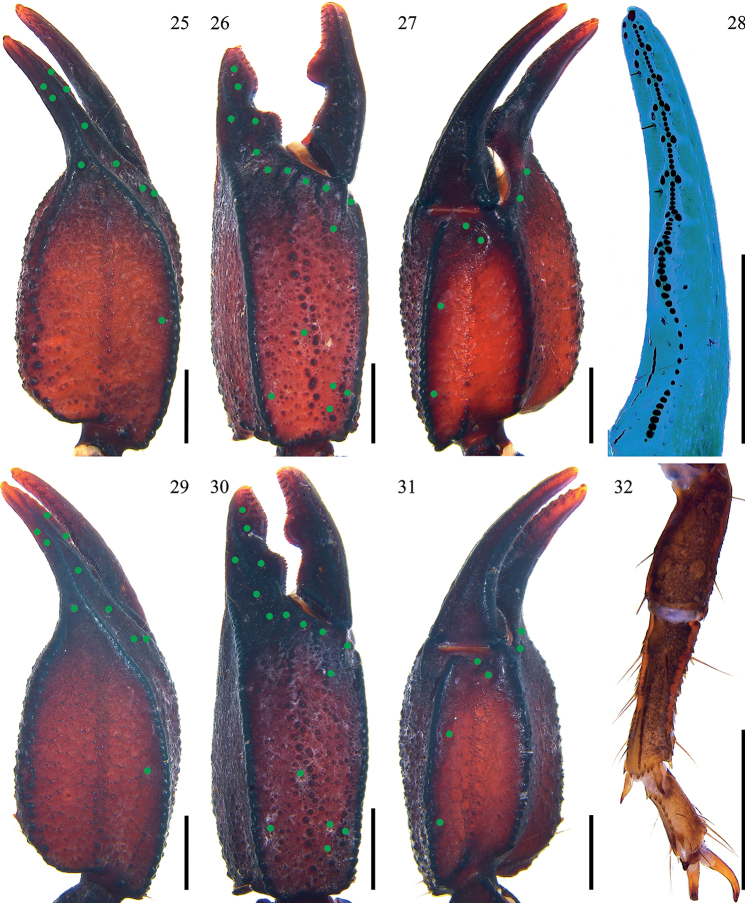
*Scorpiopsatomatus* from Lang County **25–28, 32** male (Ar.-MHBU-ScXZLX1705060101) **29–31** female (Ar.-MHBU-ScXZLX1705060102) **25–27, 29–31** chela dorsal, external, and ventral surfaces **28** dentate margin of movable finger under UV light, showing rows of granules **32** right leg I retrolateral surface. Green dots showing trichobothrial patterns of pedipalps. Scale bars: 2.0 mm.

##### Description

(based on male specimen: Ar.-MHBU-ScXZLX1705060101).

***Coloration*** (Figs 1, 2; after five years of preservation in alcohol): Carapace, reddish brown. Median and lateral ocular tubercles dark brown. Tergites and metasomal segments dark brown. Vesicle brown, with yellowish brown aculeus. Chelicerae yellow-brown, with black-brown fingers. Pedipalps dark brown. Legs brown. Tarsal claws yellowish brown. Sternum dark reddish brown. Genital operculum and sternites brown. Pectinal teeth yellowish.

##### Morphology.

***Prosoma*** (Figs 5, 6): Entire surface of carapace with fine granules. Anterior median furrow broad and shallow; lateral furrow broad and flat; posterior median furrow deep. Median ocular tubercle high and smooth, with single shallow median furrow, situated anterior to center of carapace. Lateral ocular tubercle with some large granules, three pairs of lateral ocelli, posterior smallest; smooth oval area behind lateral ocular tubercle.

***Mesosoma***: Tergites densely covered with fine granules; tergites III–VI with median carina; tergite VII with two pairs of lateral carinae (outside lateral carinae degenerated) with large granules (Fig. 12). Pectinal teeth count 10/11, fulcra present (Fig. 11). Genital operculum subtriangular with genital protruding papillae (Fig. 11). Sternum pentagonal (Fig. 11). Sternite segments III–VI entirely smooth and shiny with few setae; segment VII with four ventral carinae and few setae.

***Metasoma***: Integument coarse with few setae. Metasoma segments II–V are longer than wide; segments I–V have 10-8-8-8-7 granular carinae. All dorsal carinae granular on segment I, and gradually become strongly serrated from II–IV; segment V carinae with smaller serration dorsally and larger serration ventrally. Vesicle coarse with few setae (Fig. 15).

***Pedipalps***: Integument with smooth granules and few setae. Femur with all dorsointernal, dorsoexternal, external, ventroexternal, ventrointernal carinae granulated, and internal carinae crenulated (Fig. 16). Patella with large granules on dorsointernal, dorsoexternal, ventrointernal, ventroexternal, and external carinae; two spinoid granules present on internal surface (Figs 17–19). Trichobothrial pattern C, neobothriotaxic (Vachon, 1974); patella with 17 external trichobothria (5 *eb*, 2 *esb*, 2 *em*, 4 *est*, 4 *et*), 8 (right) and 9 (left) ventral trichobothria (Figs 18, 19). Chela with four ventral trichobothria, all carinae are granular and coalesced except the dorsal secondary, subdigital, dorsal internal, interomedian, and ventromedian carinae vestigial, movable and fixed fingers with scalloped margins, single pronounced lobe in movable finger, and a corresponding notch in fixed finger (Figs 25–28).

***Legs***: Integument coarse with few setae, except ventral aspects of coxae, trochanters, femurs, and patellae smooth. Trochanter dorsally with few granules. Femur dorsally densely granular. Patella dorsally densely granular, with dorsoexternal granular carinae. Tibiae without spurs (Fig. 32). Basitarsus with setae, spurs, and two lateral pedal spurs (Fig. 32). Tarsus ventrally with single row of spinules (Fig. 32). Tarsal ungues curved and hook-like (Fig. 32).

***Chelicerae*** (Fig. 7): Integument smooth, dorsally with an irregular pattern, ventrally with long hairs. Fixed finger of chelicera with three large triangular teeth on inner margin. Ventral of movable finger with five teeth on inner margin. Dorsal of movable finger with three teeth on inner margin.

##### Variations.

Figures of adult females are provided (Figs 3, 4, 8–10, 13, 14, 20–24, 29–31). Number (left/right) of trichobothria on the ventral surface of the pedipalp patellae: females with 9/9 (*n* = 2) and 10/9 (*n* = 1), males with 8/9 (*n* = 1) and 8/8 (*n* = 1). Number of pectinal teeth: females with 9/9 (*n* = 2) and 8/8 (*n* = 1), males with 10/11 (*n* = 1) and 11/11 (*n* = 1). Chela with an average length/width ratio of 2.3 in males (*n* = 2) and 2.5 in females (*n* = 3), male pedipalp chela fingers more strongly curved than females. All measurements are provided in Table 1. Holotype (male, not examined; Qi et al. 2005): patella with 17 external and nine ventral trichobothria, pectinal teeth count 11/11; paratype (female): some of the segments are slightly bulkier than that of the male, pectinal teeth count 9/9.

**Table 1.** Measurements (in mm) of *S.atomatus*, *S.tibetanus*, and *S.lourencoi* sp. nov.

Species Contents	*S.atomatus*		*S.tibetanus*		*S.lourencoi* sp. nov.	
Sex	Male ScXZLX 1705060101	Female ScXZLX 1705060102	Male ScXZQS 1907200101	Female ScXZQS 1907200102	Male (holotype) ScXZRKZ 2107260101	Female (paratype) ScXZRKZ 2107260102
Total length:	42.5	40.3	57.3	52.7	46.7	47.3
Carapace:						
– Length	6.6	6.8	7.7	7.3	6.5	6.7
– Anterior width	4.1	4.1	3.9	3.9	3.6	3.7
– Posterior width	6.9	7.3	7.8	7.6	6.7	6.9
Mesosomal segments:						
– Length	11.6	10.9	14.7	15.0	14.1	14.9
Metasomal segment I:						
– Length	2.5	2.4	3.6	3.0	2.7	2.7
– Width	2.5	2.4	4.1	3.6	3.0	2.8
– Depth	2.1	2.0	2.9	2.5	2.1	2.0
Metasomal segment II:						
– Length	2.9	2.6	4.3	3.7	3.2	3.1
– Width	2.1	2.1	3.6	3.2	2.7	2.6
– Depth	2.0	2.0	3.4	3.0	2.3	2.1
Metasomal segment III:						
– Length	3.0	2.8	5.0	4.4	3.7	3.7
– Width	2.1	2.0	3.6	3.0	2.7	2.6
– Depth	2.2	2.1	3.2	2.7	2.3	2.1
Metasomal segment IV:						
– Length	3.5	3.3	5.5	5.2	3.9	3.8
– Width	2.0	1.9	3.2	2.8	2.4	2.4
– Depth	1.9	1.9	2.9	2.7	2.2	2.1
Metasomal segment V:						
– Length	6.0	5.6	8.3	7.4	6.1	5.9
– Width	2.0	1.9	2.6	2.1	2.1	2.0
– Depth	1.9	1.7	2.7	2.2	1.8	1.8
Telson:						
– Length	6.4	5.9	8.2	7.3	6.5	6.5
– Width	2.4	2.4	3.3	2.8	2.7	2.2
– Depth	2.4	2.2	3.1	2.8	2.5	2.2
Pedipalp femur:						
– Length	5.6	5.6	5.4	4.8	4.9	4.8
– Width	2.5	2.6	2.6	2.3	2.5	2.4
– Depth	1.5	1.5	2.2	2.1	1.9	1.8
Pedipalp patella:						
– Length	5.3	5.7	6.2	5.8	5.2	5.4
– Width	3.2	3.3	2.7	2.6	2.7	2.1
– Depth	2.4	2.2	2.6	2.6	2.5	2.4
Chela:						
– Length	11.3	11.3	11.5	10.9	9.9	10.4
– Width (manus)	4.9	4.5	5.6	5.3	5.3	4.4
– Depth (manus)	3.1	2.9	4.0	3.9	3.6	3.3
Movable finger:						
– Length	4.3	3.8	4.6	4.4	3.6	3.1
Pectinal teeth	10/11	9/9	7/7	5/4	8/9	7/8

##### Distribution.

China (Xizang) (Fig. 97).

##### Remarks.

Body size is an important feature in distinguishing between the *Scorpiops* species. In China, *S.atomatus* (Xizang), *Scorpiopsjendeki* Kovařík, 2000 (Yunnan), *Scorpiopslhasa* Di & Zhu, 2009 (Xizang), and *Scorpiopstaxkorgan* Lourenço, 2018 (Xinjiang) are undoubtedly small species. In this work, we tried to separate the small-type species: usually < 50 mm, such as *S.atomatus*; the medium-sized species, usually 50–70 mm, such as *S.pococki* and *S.langxian*; and the larger species, usually > 70 mm, including *S.luridus* and *S.ingens* Yin, Zhang, Pan, Li & Di, 2015.

Di et al. (2011) thought that *S.atomatus* should be excluded from the *S.hardwickii* complex previously proposed by Kovařík and Ahmed (2009) due to the following reasons: (i) pectinal teeth count is 9–11 in *S.atomatus* and 4–8 in *S.hardwickii*; (ii) ventral trichobothria on patella are nine in *S.atomatus* and 6–8 in *S.hardwickii*; (iii) fulcra are present in *S.atomatus* but absent in *S.hardwickii*. In addition, *S.atomatus* has clearly thinner chela than *S.pococki* and *S.langxian.*

The most important morphological difference is that the body length of *S.tibetanus* holotype is 60.4 mm, and Kovařík (2000) recorded *S.tibetanus* as 50–65 mm; although there may be different measurement methods used by different authors, it suggested *S.tibetanus* significantly longer than *S.atomatus*. Here, we reaffirm the validity of *S.atomatus* based on newly collected materials.

#### ﻿ Scorpiops tibetanus

Taxon classificationAnimaliaScorpionesEuscorpiidae

Hirst, 1911

6468ABCA-5F45-5BF9-AF31-BF2558DAC162

Figs 33–36 , 37–46 , 47–56 , 57–64 , Table 1

Scorpiops tibetanus Hirst, 1911: 472–473; Kovařík 2000: 196, figs 47, 68, 69, tab. 1–3; Fet 2000: 495; Kovařík et al. 2020: 126, figs 46, 143, 239–240, 799, tab. 9.
Scorpiops pococki Qi, Zhu & Lourenço, 2005: 14, figs 47–61; Di et al. 2013: 72, 75, figs 64–84, tab. 3; Di et al. 2014: 12.

##### Type locality.

China, Xizang, Tsangpo Valley, Chaksam Ferry.

##### Material examined.

1 male and 1 female, China, Xizang, Lasa City (Lhasa City), Qushui County (Chushur County), Caina Town (Saena Town), 20/7/2019, Zhiyong Di leg, (Ar.-MHBU-ScXZQS1907200101, Ar.-MHBU-ScXZQS1907200102); 1 male and 2 females, China, Xizang, Shannan City (Lhoka City), Jiacha County (Gyaca County), Jiacha Town (Gyaca Town), 12/8/2021, Zhiyong Di leg, (Ar.-MHBU-ScXZJC21081206, 01–03).

##### Diagnosis.

Adult body length 50–57 mm. Base color uniformly reddish black. Patella of pedipalp with 17 (5 *eb*, 2 *esb*, 2 *em*, 4 *est*, 4 *et*) external and 6–8 (usually seven) ventral trichobothria. Chelal trichobothria *Eb_3_* is located in proximal half of manus between trichobothria *Dt* and *Db.* Chela with four ventral trichobothria. Chela with an average length/width ratio of 2.0 in both sexes, pedipalp movable finger with ca. four or five ID, 10–25 IAD, 45–62 MD, and eight or nine OD present, chela fingers on adult males and females scalloped, usually more strongly in males. Pectinal teeth count 4–7, fulcra absent. Pectinal with two marginal and one middle lamellae. Telson bulbous and granulate, annular ring present.

##### Description

(based on male specimen: Ar.-MHBU-ScXZQS1907200101).

**Figures 33–36.**
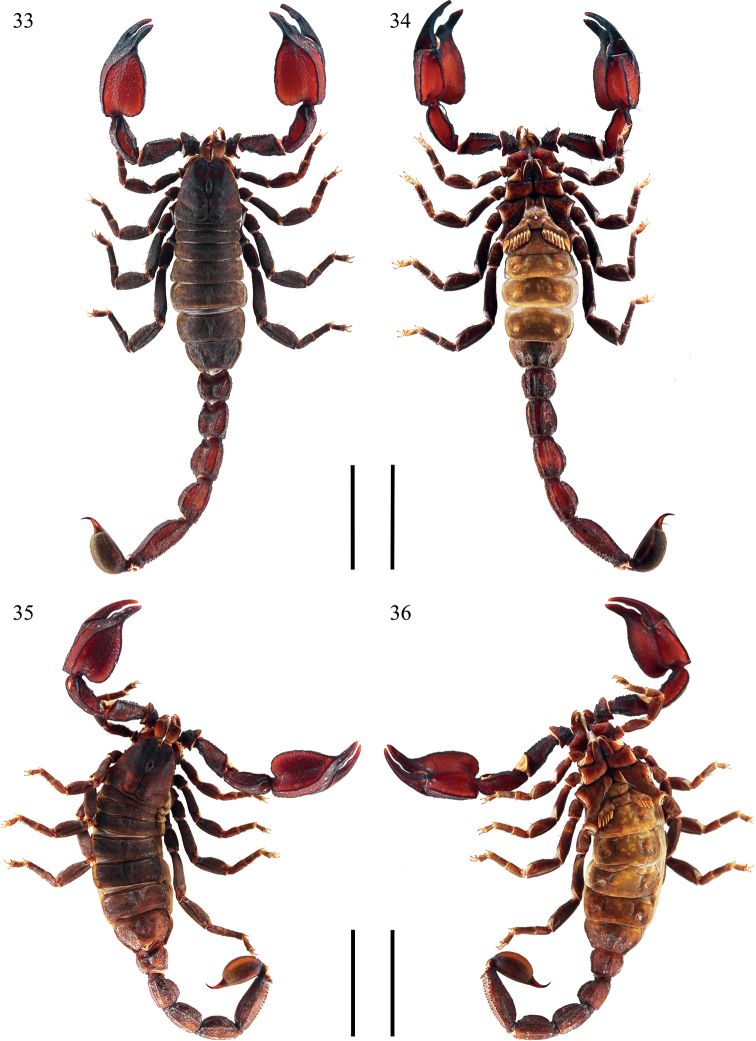
*Scorpiopstibetanus* from Qushui County **33, 34** male (Ar.-MHBU-ScXZQS1907200101), dorsal and ventral views **35, 36** female (Ar.-MHBU-ScXZQS1907200102), dorsal and ventral views. Scale bars: 12.0 mm.

***Coloration*** (Figs 33, 34; after three years of preservation in alcohol): Carapace reddish black. Median and lateral ocular tubercles dark brown. Tergites and metasomal segments dark brown. Vesicle dark brown, with dark brown aculeus. Chelicerae unevenly dark brown and fingers uniformly dark reddish. Pedipalps dark reddish brown. Legs dark brown. Tarsal claws brown. Sternum reddish brown. Genital operculum and sternites brown. Pectinal teeth light brown.

##### Morphology.

**Figures 37–46.**
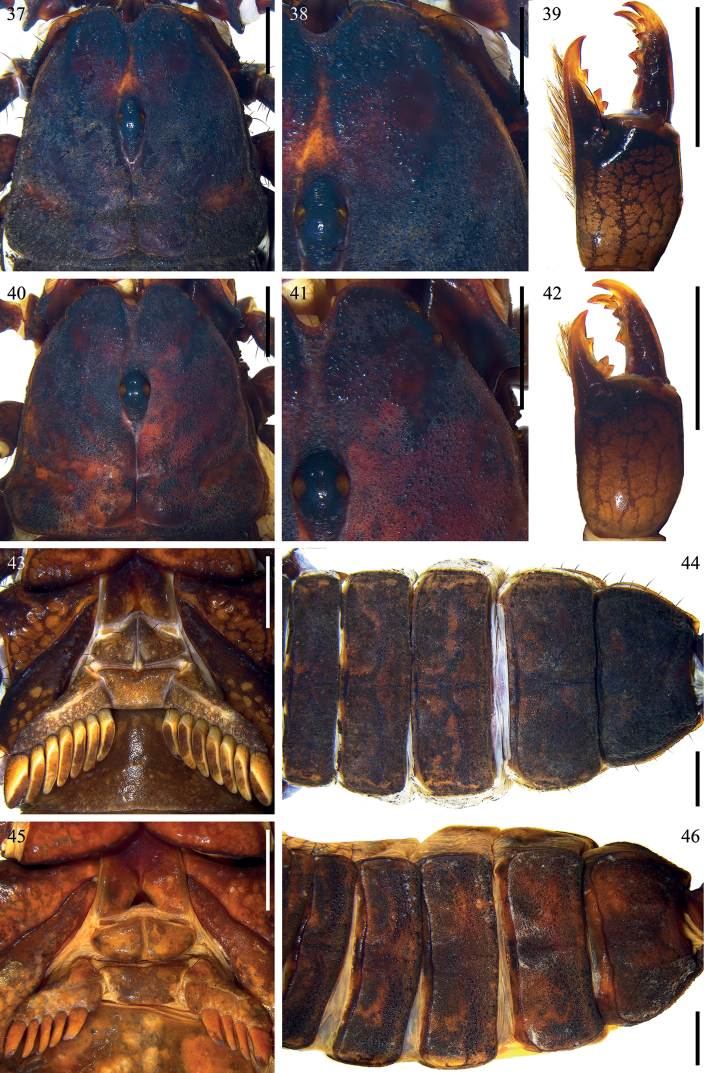
*Scorpiopstibetanus* from Qushui County **37, 38, 39, 43, 44** male (Ar.-MHBU-ScXZQS1907200101) **40, 41, 42, 45, 46** female (Ar.-MHBU-ScXZQS1907200102) **37, 40** carapace **38, 41** eyes and nearby area **39, 42** chelicera dorsal surface **43, 45** sternum, genital operculum and pectines **44, 46** tergites. Scale bars: 2.0 mm.

***Prosoma*** (Figs 37, 38): Integument coarse, carapace with dense, fine granules; anterior median furrow broad and deep; lateral furrow broad; posterior median furrow broad and deep. Median eyes situated anteriorly compared to center of carapace; three pairs of lateral ocelli with posterior-most the smallest. Median ocular tubercle with granules and median furrow. Lateral ocular tubercle with some coarse granules around lateral eyes.

***Mesosoma***: Tergites densely covered with fine granules, tergites II–VII with median carina barely visible at first and gradually becomes distinct; tergite VII with two pairs of lateral carinae with large granules present only on posterior half (Fig. 44). Pectinal teeth count 7/7, fulcra absent (Fig. 43). Genital operculum subtriangular with genital papillae protruding (Fig. 43). Sternum pentagonal (Fig. 43). Sternite segments III–VI entirely smooth and shiny with few setae; segment VII with four ventral carinae and few setae.

**Figures 47–56.**
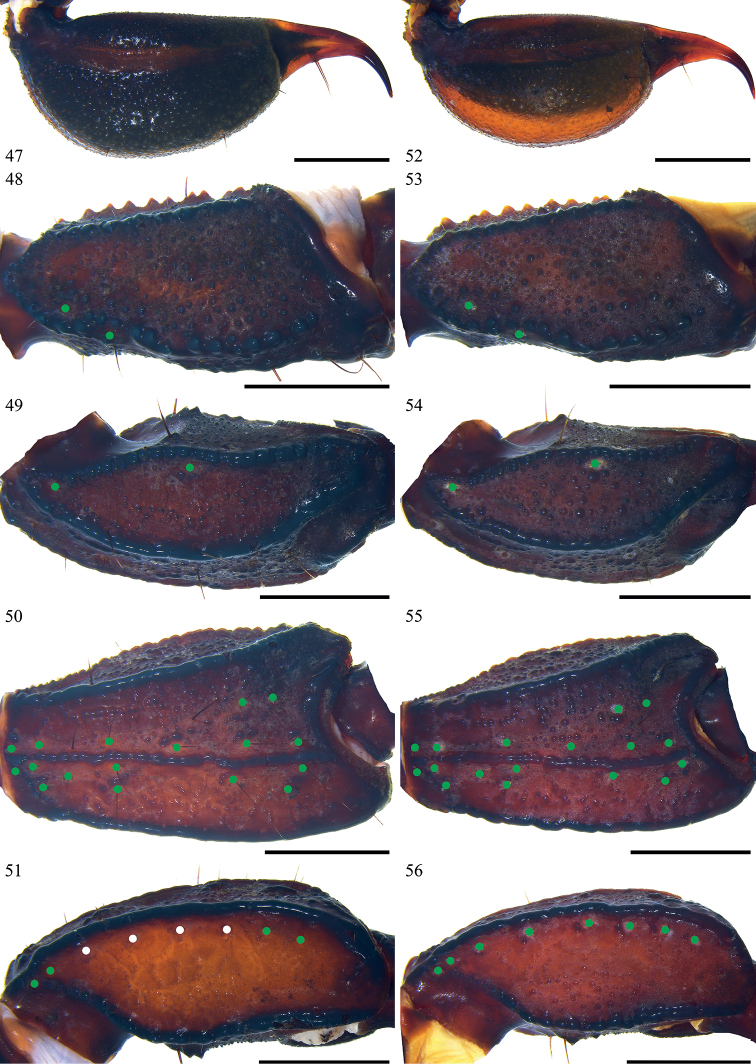
*Scorpiopstibetanus* from Qushui County **47–51** male (Ar.-MHBU-ScXZQS1907200101) **52–56** female (Ar.-MHBU-ScXZQS1907200102) **47, 52** telson, lateral surface **48, 53** femur dorsal surface **49–51, 54–56** patella dorsal, external, and ventral surfaces. Green dots showing trichobothrial patterns of pedipalps (the white dots representing dysplasia caused trichobothrial absence). Scale bars: 2.0 mm.

***Metasoma***: Integument coarse, segments II–V longer than wide; segments I–V with respectively 10-8-8-8-8 granular carinae; segment V with pair of vestigial lateral carinae; all ventral carinae crenulated, gradually becoming more strongly crenulated. Vesicle with dense granules and few setae (Fig. 47).

**Figures 57–64.**
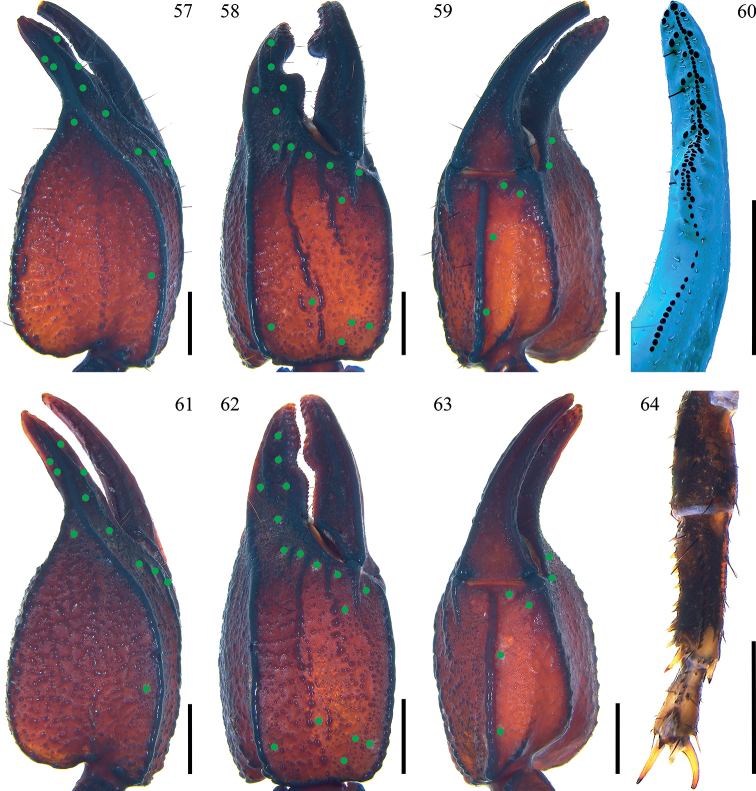
*Scorpiopstibetanus* from Qushui County and Jiacha County **57–59, 64** male (Ar.-MHBU-ScXZQS1907200101) **61–63** female (Ar.-MHBU-ScXZQS1907200102) **60** male (Ar.-MHBU-ScXZJC2108120601, from Jiacha County) **57–59, 61–63** dorsal, external, and ventral surfaces of chela **60** dentate margin of movable finger under UV light, showing rows of granules **64** right leg I retrolateral surface. Green dots showing trichobothrial patterns of pedipalps. Scale bars: 2.0 mm.

***Pedipalps***: Integument smooth with smooth granules and few setae. Femur with dorsointernal, dorsoexternal, external, ventroexternal, ventrointernal carinae granulated, and internal carinae crenulated (Fig. 48). Patella with large granules on dorsointernal, dorsoexternal, ventrointernal, ventroexternal, and smooth external carinae; two spinoid granules present on internal surface (Figs 49–51). Trichobothrial pattern C, neobothriotaxic (Vachon, 1974); patella with 17 external trichobothria (5 *eb*, 2 *esb*, 2 *em*, 4 *est*, 4 *et*), 4 (right, dysplastic) and 7 (left) ventral trichobothria (Figs 50, 51). Chela with granules forming the indistinct reticulated pattern, ventral with four trichobothria, all carinae are granular and coalesced except the subdigital, dorsal internal, interomedian, and ventromedian carinae vestigial; movable and fixed fingers with scalloped margins, a pronounced lobe in movable finger and corresponding notch in fixed finger (Figs 57–60).

***Legs***: Integument coarse with few setae. Trochanter dorsal surface with few granules. Femur dorsal surface densely granular. Patella dorsal surface densely granular, with dorsoexternal and dorsal granular carinae. Tibiae without spurs (Fig. 64). Basitarsus with more setae, spurs, and two lateral pedal spurs (Fig. 64). Tarsus ventrally with single row of spinules (Fig. 64). Tarsal ungues curved and hook-like (Fig. 64).

***Chelicerae*** (Fig. 39): Dorsally with irregular pattern, ventrally with long hairs. Fixed finger of chelicera with three large triangular teeth on inner margin; ventral of movable finger with five teeth on inner margin, dorsal of movable finger with four teeth on inner margin.

##### Variation.

Figures of adult females are provided (Figs 35, 36, 40–42, 45, 46, 52–56, 61–63). Number (left/right) of trichobothria on the ventral surface of the pedipalp patellae: females with 8/8 (*n* = 1), 7/7 (*n* = 1), and 6/6 (*n* = 1), males with ?/7 (*n* = 1) and 7/7 (*n* = 1). Number of pectinal teeth: females with 5/4 (*n* = 2) and 6/5 (*n* = 1), males with 7/7 (*n* = 2). Chela with an average length/width ratio of 2.0 in males (*n* = 2) and 2.0 in females (*n* = 3), male pedipalp chela fingers stronger curved than females. The measurements are provided in Table 1. Holotype (male, not examined; Kovařík, 2000): body length 60.4 mm, patella with eight ventral trichobothria, pectinal teeth count seven or eight. Holotype (male) of *S.pococki* (= *S.tibetanus*): patella with 17 external and eight ventral trichobothria, pectinal teeth count 8/7; paratype (female) of *S.pococki* (= *S.tibetanus*): pectinal teeth count 6/6, telson is smaller than that of male in Qi et al. (2005).

##### Distribution.

China (Xizang) (Fig. 97).

##### Remarks.

Hirst (1911) erected *S.tibetanus* based on a male specimen from “Tsangpo Valley, Chaksam Ferry”, for which the species information is brief, and no figures provided.

The type locality is therefore the most crucial information on the species. Followed the Internet and literature information for “Chaksam Ferry”: “Chaksam” means “iron bridge”, located in Dagar village and under the Quwori Mountain, Qushui County. It was created by the famous bridge designer, Tangdongjiebu, and is the first cable bridge across the Brahmaputra River, completed in 1420 or 1430. “Chaksam Ferry” subsequently became a famous place, also called “Qushui Ferry” or “Daga Ferry” in ancient times (Fig. 97). However, with the disappearance of the “iron bridge” and the construction of Qushui Bridge (Qushuidaqiao) near where the ancient “iron bridge” used to be in 1966, “Chaksam Ferry” has now been replaced by Qushui Bridge and is the probable type locality of *S.tibetanus*.

In recent years, we found *S.atomatus* distributed near the “iron bridge”, and found *S.pococki* 28 km away from this “iron bridge” (Fig. 97).

The list of taxa included in the *S.hardwickii* “complex” proposed by Kovařík and Ahmed (2009) included *S.tibetanus*. Kovařík (2000) examined the holotype (male) of *S.tibetanus* and recorded some important information: “length 60.4 mm, ventral trichobothria on the patella number 8, and pectinal teeth number 7 or 8”. Kovařík (2000) concluded that the characters of *S.tibetanus* were “length 50–65 mm, ventral trichobothria on the patella number 7–10 (usually 9, in one young of 37 specimens, 7 on one side), pectinal teeth number 5–11”. In his revision, the new localities of *S.tibetanus* included Lhasa, Shigatse, and Kambu batsi. Kovařík et al. (2020) proposed *S.pococki* as a junior synonym of *S.tibetanus* and provided figures of *S.tibetanus* (one male identified as “*S.pococki*” and donated by Di).

During several surveys in Shigatse and the surrounding counties, we found *S.lourencoi* sp. nov. and *S.luridus.* The body length of *S.lourencoi* sp. nov. is no more than 50 mm, while *S.luridus* is a distinctive species. *Scorpiopspococki* (from Chaksam Ferry 28 km away) has the diagnosis as followed: fulcra absent, body length ~ 55 mm, the ventral trichobothria on the patella number seven or eight, and the pectinal teeth number 4–7. *Scorpiopspococki* fits the characteristics of *S.tibetanus*, and we agree that *S.pococki* is a synonym of *S.tibetanus*.

#### ﻿ Scorpiops lourencoi sp. nov.

Taxon classificationAnimaliaScorpionesEuscorpiidae

BC7F71B7-CFFB-5F09-8532-5E32A1503413

https://zoobank.org/92D21DC0-794E-4D76-B2F8-28AB9D88520B

Figs 65–68 , 69–78 , 79–88 , 89–96 , Table 1

Scorpiops tibetanus Di et al., 2013: 75, 77, 80, 81, 83, 85, figs 102–118, tab. 2.

##### Type material.

**Male holotype**, China, Xizang, Rikaze City (Shigatse City), 26/7/2021, Zhiyong Di leg, (Ar.-MHBU-ScXZRKZ2107260101); 3 male and 3 female paratypes (Ar.-MHBU-ScXZRKZ21072601, 02–06; Ar.-MHBU-ScXZRKZ2107270501), same location data as holotype.

##### Diagnosis.

*Scorpiopslourencoi* sp. nov. differs from all other species in the genus based on the following combination of characters: reddish black color, length 45–50 mm; patella of pedipalp with 17 (5 *eb*, 2 *esb*, 2 *em*, 4 *est*, 4 *et*) external and eight or nine (usually nine) ventral trichobothria. Chelal trichobothria *Eb_3_* located in proximal half of manus between trichobothria *Dt* and *Db*, chela with four ventral trichobothria; chela with an average length/width ratio of 1.9 in males (*n* = 4 adults) and 2.4 in females (*n* = 3 adults); pedipalp movable finger with ca. four or five ID, 10–14 IAD, 44–45 MD, and seven or eight OD present; pedipalp chelal fingers on adult males and females scalloped, usually more strongly in male. Pectinal teeth count 8–11 (usually 10) in four males and seven or eight (usually seven) in three females, fulcra present; pectinal with three marginal and five or six middle lamellae. Telson bulbous and granulate, annular ring present.

##### Etymology.

Patronym in honor of Prof. Wilson R. Lourenço, who significantly contributed to scorpion research.

##### Description

(based on holotype: Ar.-MHBU-ScXZRKZ2107260101).

**Figures 65–68.**
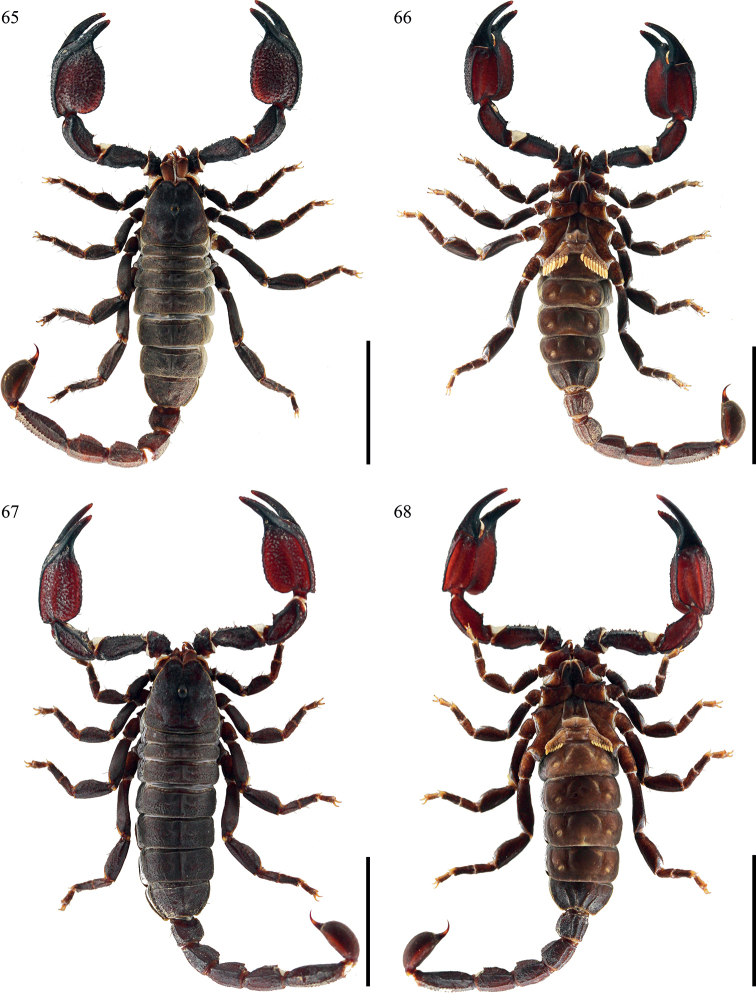
*Scorpiopslourencoi* sp. nov. from Rikaze City **65, 66** male (Ar.-MHBU-ScXZRKZ2107260101), dorsal and ventral views **67, 68** female (Ar.-MHBU-ScXZRKZ2107260102), dorsal and ventral views. Scale bars: 12.0 mm.

***Coloration*** (Figs 65, 66; after one year of preservation in alcohol): Carapace, dark red-brown. Median and lateral ocular tubercles dark brown. Tergites and metasomal segments dark red-brown to dark brown. Vesicle dark brown, with brown aculeus. Chelicerae dark brown; with the fingers dark brown and gradually lighter toward the tip. Pedipalps dark red-brown. Legs dark brown. Tarsal claws yellowish brown. Sternum, genital operculum, and sternites yellowish brown. Pectinal teeth yellowish.

##### Morphology.

**Figures 69–78.**
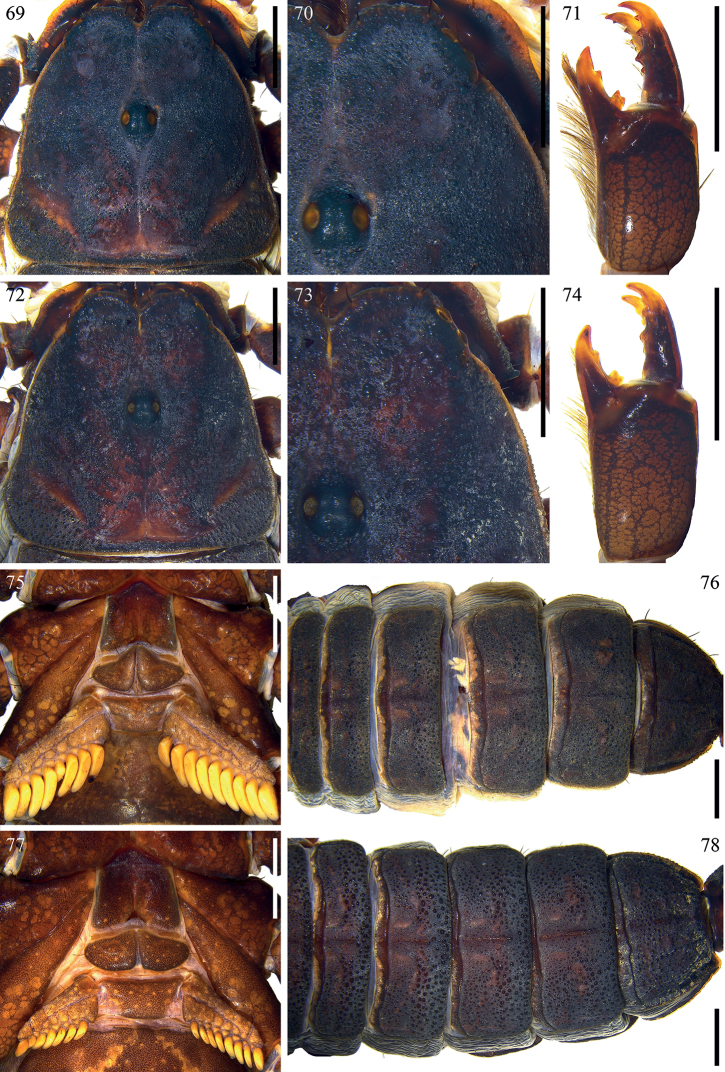
*Scorpiopslourencoi* sp. nov. from Rikaze City **69, 70, 71, 75, 76** male (Ar.-MHBU-ScXZRKZ2107260101) **72, 73, 74, 77, 78** female (Ar.-MHBU-ScXZRKZ2107260102) **69, 77** carapace **70, 73** eyes and nearby area **71, 74** chelicera dorsal surface **75, 77** sternum, genital operculum, and pectines **76, 78** tergites. Scale bars: 2.0 mm.

***Prosoma*** (Figs 69, 70): Carapace with dense, coarse granules; shallow anterior median furrow; broad and flat lateral furrow; posterior median furrow broad and deep. Median ocular tubercle high and coarse, with a shallow median furrow, median eyes situated anteriorly compared to center of carapace; three pairs of lateral ocelli, posterior smallest, some large granules near lateral ocelli, and presence of smooth oval area behind the lateral ocular tubercle.

***Mesosoma***: Integument coarse. Tergites sparsely covered with large coarse granules, posterior part of tergites with larger granules; tergites III–VI with a median carina; tergite VII with two pairs of lateral carinae (outside lateral carinae degenerated) (Fig. 76). Sternum quinquangular with few setae (Fig. 75). Genital operculum subtriangular with genital papillae protruding (Fig. 75). Pectinal teeth count 8/9, fulcra present (Fig. 75). Sternites, segments III–VI are smooth and shiny with few setae, segment VII with four smooth carinae of big granules and few setae.

**Figures 79–88.**
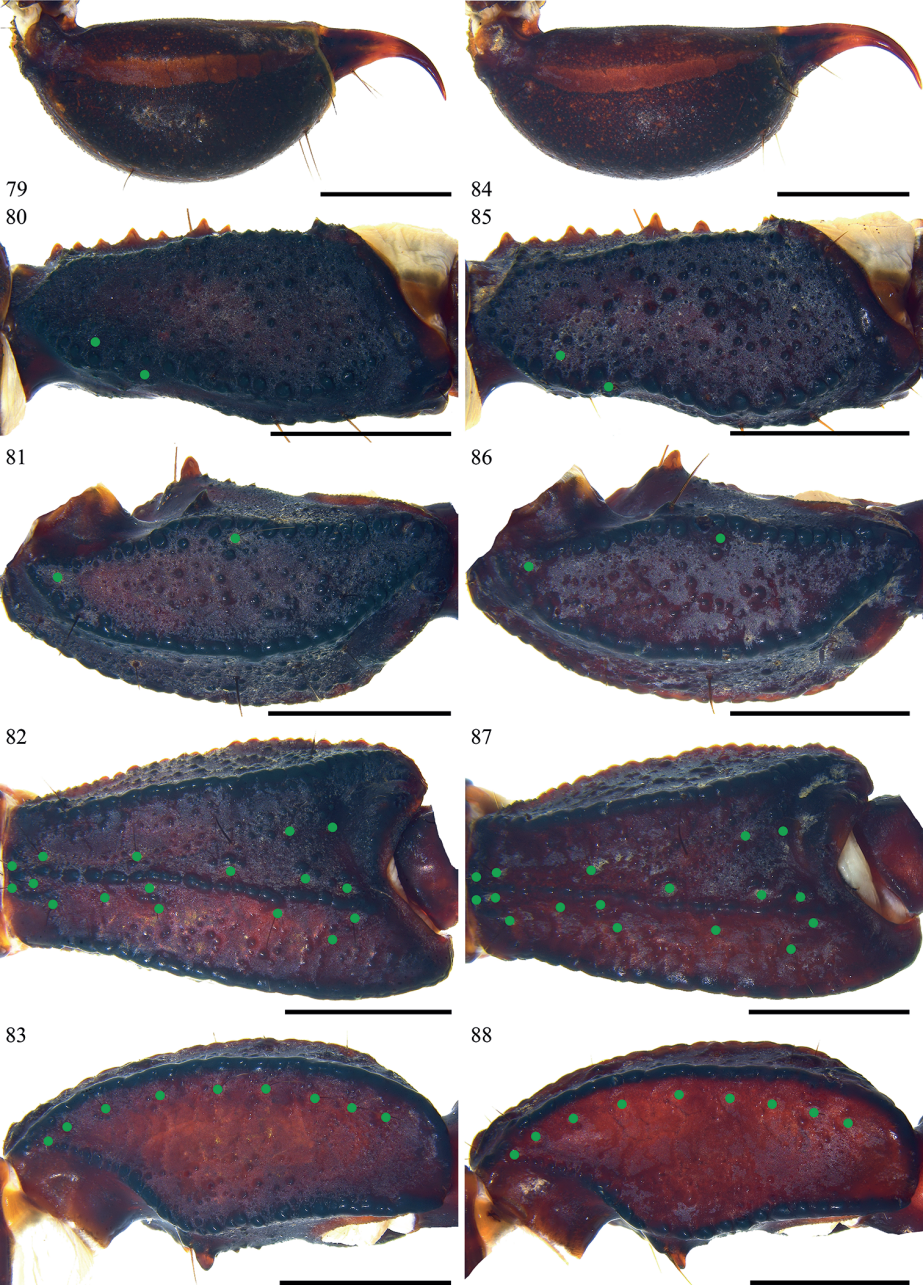
*Scorpiopslourencoi* sp. nov. from Rikaze City **79–83** male (Ar.-MHBU-ScXZRKZ2107260101) **84–88** female (Ar.-MHBU-ScXZRKZ2107260102) **79, 84** telson, lateral surface **80, 85** femur dorsal surface **81–83, 86–88** patella dorsal, external, and ventral surfaces. Green dots showing trichobothrial patterns of pedipalps. Scale bars: 2.0 mm.

***Metasoma***: Integument coarse with few setae. Segments II–V longer than wide; segments I–V with respectively 10-8-8-8-7 granular carinae, segments II–IV all dorsal carinae gradually become strongly serrated; segment V carinae with smaller granules dorsally and larger serration ventrally. Vesicle with few setae and granules (Fig. 79).

**Figures 89–96.**
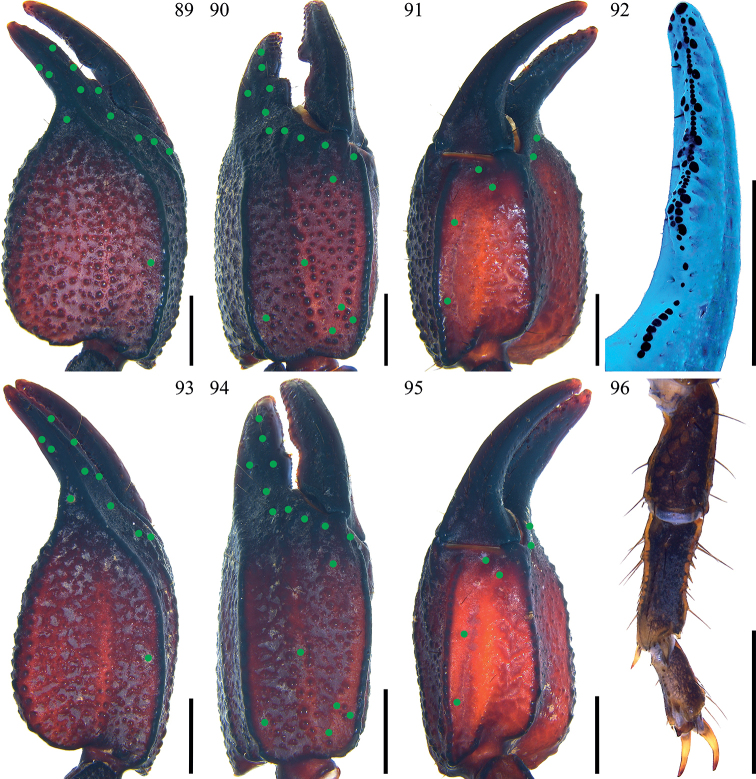
*Scorpiopslourencoi* sp. nov. from Rikaze City **89–92, 96** male (Ar.-MHBU-ScXZRKZ2107260101) **93–95** female (Ar.-MHBU-ScXZRKZ2107260102) **89–91, 93–95** chela dorsal, external, and ventral surfaces **92** dentate margin of movable finger under UV light, showing rows of granules **96** right leg I retrolateral surface. Green dots showing trichobothrial patterns of pedipalps. Scale bars: 2.0 mm.

***Pedipalps***: Integument with smooth granules and few setae, especially the granules are larger on dorsal and external surfaces of chelae. Femur with external, dorsointernal, dorsoexternal, ventrointernal, ventroexternalcarinae granulated, and internal carinae crenulated (Fig. 80). Patella with dorsointernal, ventrointernal, ventroexternal, and external carinae with smooth granules; two spinoid granules present on the internal aspect, the ventral internal spinoid granule being much larger than the dorsointernal one (Figs 81–83). Trichobothrial pattern C, neobothriotaxic (Vachon, 1974); patella with 17 external trichobothria (5 *eb*, 2 *esb*, 2 *em*, 4 *est*, 4 *et*), 9 (right) and 9 (left) ventral trichobothria (Figs 82, 83). Chela very thick, with four ventral trichobothria, all carinae are granular and coalesced except the dorsal secondary, subdigital, dorsal internal, interomedian, and ventromedian carinae vestigial; fingers scalloped, with a pronounced lobe in the movable finger and a corresponding notch in the fixed finger (Figs 89–92).

***Legs***: Integument coarse with few setae except ventral aspects of coxae, trochanters, femurs, and patellae smooth. Trochanter dorsal with few granules and few setae. Femur dorsal with few granules. Patella internally with a dentate carina. Tibiae without spurs (Fig. 96). Basitarsus with spurs and two lateral pedal spurs (Fig. 96). Tarsus ventrally with a row of spinules (Fig. 96). Tarsal ungues curved and hook-like (Fig. 96).

***Chelicerae*** (Fig. 71): Integument smooth and shiny, dorsal with irregular pattern, ventrally with long hairs. Fixed finger of chelicera with three large triangular teeth on inner margin; ventral of movable finger with six teeth on inner margin, dorsal of movable finger with four teeth on inner margin.

##### Variation.

Feature figures of adult females are provided (Figs 67, 68, 72–74, 77, 78, 84–88, 93–95). Number (left/right) of trichobothria on the ventral surface of the pedipalp patellae: females with 9/9 (*n* = 2) and 8/9 (*n* = 1), males with 9/9 (*n* = 4). Number of pectinal teeth: females with 7/7 (*n* = 2) and 7/8 (*n* = 1), males with 8/9 (*n* = 1), 10/10 (*n* = 2), and 10/11 (*n* = 1). Chela with an average length/width ratio of 1.9 in males (*n* = 4) and 2.4 in females (*n* = 3), male pedipalp chela fingers stronger curved than females, lobe and corresponding notch reduced in females. Measurements provided in Table 1. One female specimen (mistakenly identified as *S.tibetanus* in Di et al. (2013); not examined): body length 45.2 mm, patella with nine ventral trichobothria, pectinal teeth count 7/7.

##### Habitat.

Found under stones in dry mountain boscage in Shigatse City, ~ 3889 m elevation.

##### Distribution.

**Figure 97.**
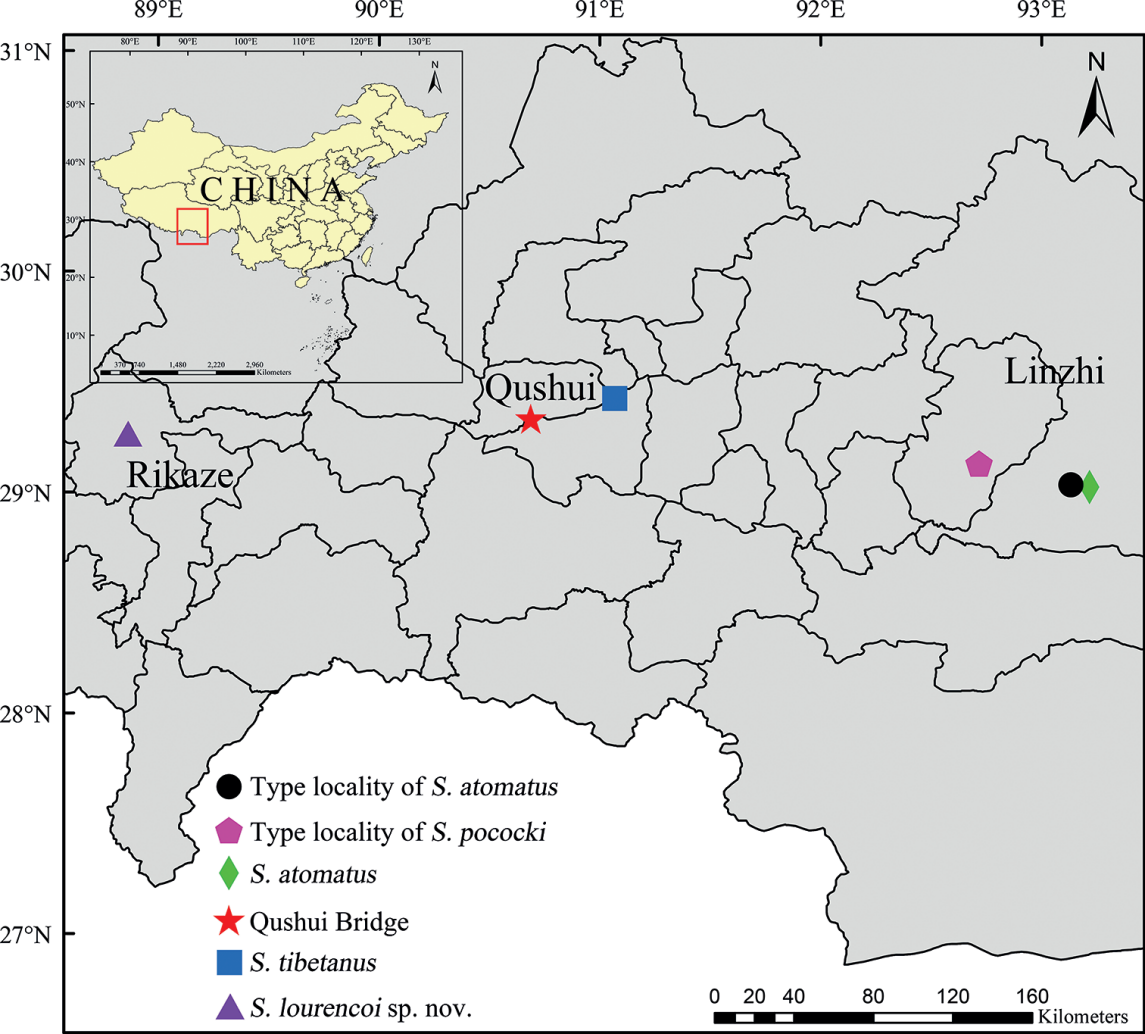
Distribution of *S.atomatus* (circle and diamond), *S.tibetanus* (pentagon and square), *S.lourencoi* sp. nov. (triangle), and the location of Qushui Bridge and Chaksam Ferry (star) in Xizang, China.

Rikaze City, Xizang, China (Fig. 97).

##### Remarks.

*Scorpiopsatomatus* is similar to the new species, but can be readily distinguished based on the following combination of characters: (i) the pectinal teeth count 8–11 (with 10–11 (usually 11) in males and eight or nine (usually nine) in females) in *S.atomatus*, while there are 7–11 (with 8–11 (usually 10) in males and seven or eight (usually seven) in females) in *S.lourencoi* sp. nov. (ii) length of adults 40–45 mm in *S.atomatus*, while the length of adults 45–50 mm in *S.lourencoi* sp. nov. (iii) chela with an average length/width ratio of 2.3 in males (*n* = 2 adults) and 2.5 in females (*n* = 3 adults) in *S.atomatus*, while 1.9 in males (*n*= 4 adults) and 2.4 in females (*n* = 3 adults) in *S.lourencoi* sp. nov. (iv) chela surface with small granules in *S.atomatus*, while large granules in *S.lourencoi* sp. nov.

## ﻿Discussion

Kovařík (2000), Kovařík and Ahmed (2009), and Kovařík et al. (2020) examined the type specimen of *S.tibetanus*, however, they provided confusing information that led to *S.pococki* and *S.atomatus* being considered synonyms of *S.tibetanus*. Influenced by this, Qi et al. (2005) incorrectly erected *S.pococki*. The precise information on Chaksam and Chaksam Ferry was not easily available in 2005, and the revision of the family Scorpiopidae by Kovařík (2000) was undoubtedly the most important basis for Qi et al. (2005). Di et al. (2011, 2013, 2014), Di and Qiao (2020), Li et al. (2016), and Yin et al. (2015) also relied on Kovařík’s (2000) information when reviewing the *Scorpiops* species from China. This led to mistakes in the keys related to *Scorpiops* from China by Di et al. (2011, 2013, 2014), Di and Qiao (2020), Li et al. (2016), and Yin et al. (2015). Those keys of *Scorpiops* species from China should be corrected and the new revised key is presented below.

### ﻿Updated key to Chinese species of the genus *Scorpiops* with chelal trichobothria *Eb_3_* located in the proximal half of the manus between trichobothria *Dt* and *Db* (modified from Di and Qiao 2020)

**Table:** 

1	Pedipalp chela fingers with non-scalloped (nearly straight) margins in both sexes	**2**
–	Pedipalp chela fingers with scalloped margins in male adults	**4**
2	Chela length-to-width ratio > 3.0	**3**
–	Chela length-to-width ratio < 3.0	***S.jendeki***
3	Total length 40.0–58.0 mm, chela length-to-width ratio ~ 3.3–3.5	***S.leptochirus***
–	Total length 35.2 mm (male holotype), chela length-to-width ratio ~ 3.2	***S.taxkorgan***
4	Manus length-to-width ratio visibly > 1	**5**
–	Manus with similar length and width	**12**
5	Total length > 61.0 mm usually	**6**
–	Total length < 61.0 mm usually	**8**
6	Red brown, ventral patella of pedipalps with 7 (rarely 6 or 8) trichobothria	***S.petersii***
–	Lighter than red brown	**7**
7	Ventral patella of pedipalps with 7 or 8 trichobothria, pectinal teeth count 7 in males and 6 in females	***S.songi***
–	Ventral patella of pedipalps with 9 trichobothria, pectinal teeth count 9/10 in male holotype and 8 in female paratype	***S.luridus***
8	Dorsally flat manus of pedipalps and chela of both sexes, with length/width ratio: 2.1–2.2 (~ 2.1 in males and 2.2 in females), total length 40.0–50.0 mm in adults	***S.margerisonae***
–	Dorsally round manus of pedipalps or at least the chela of one sex, with length-to-width ratio > 2.2 or total length > 50.0 mm	**9**
9	Body length ~ 45.0 mm–61.0 mm	**10**
–	Body length < 40.0 mm	**11**
10	Patella of pedipalp with 17 (5 *eb*, 2 *esb*, 2 *em*, 4 *est*, 4 *et*) external trichobothria	***S.lourencoi* sp. nov.**
–	Patella of pedipalp with 18–20 (5 *eb*, 2 *esb*, 2 *em*, 5 *est*, 4–6 *et*) external trichobothria	***S.wrzecionkoi***
11	Chela of pedipalp length-to-width ratio ~ 2.6–3.0	***S.lhasa***
–	Chela of pedipalp length-to-width ratio < 2.5	***S.atomatus***
12	Yellow-brown color, length of adults > 70.0 mm	***S.ingens***
–	Red-brown to red-black color, length of adults < 65.0 mm	***S.hardwickii* complex (*S.hardwickii*, *S.jingshanensis*, *S.langxian*, *S.tibetanus*)**

## Supplementary Material

XML Treatment for Scorpiops

XML Treatment for Scorpiops atomatus

XML Treatment for Scorpiops tibetanus

XML Treatment for Scorpiops lourencoi
